# The Roles of Three Types of Knowledge and Perceived Uncertainty in Explaining Risk Perception, Acceptability, and Self-Protective Response—A Case Study on Endocrine Disrupting Surfactants

**DOI:** 10.3390/ijerph15020296

**Published:** 2018-02-08

**Authors:** Hien Ho, Tsunemi Watanabe

**Affiliations:** 1 Graduate School of Engineering, Kochi University of Technology, Tosayamada, Kami City, Kochi 782-8502, Japan; thanhhien119.ho@gmail.com; 2 School of Economics and Management, Kochi University of Technology, 2-22 Eikokuji, Kochi City, Kochi 780-8515, Japan

**Keywords:** risk perception, risk acceptability, self-protective response, knowledge, perceived uncertainty, endocrine disrupting surfactants (EDSs), health behavior

## Abstract

The ubiquitous surfactants nonylphenol (NP) and its ethoxylates (NPEOs), which are known as endocrine disrupters, have appeared in the lists of restricted chemical substances, monitoring programs, and environmental quality standards of many countries due to their adverse effects. Recent studies have reported alarming levels of NP, as the final metabolite of NPEOs, in Vietnamese urban waters, whilst response to this issue is negligible. With the aim of addressing how the public perceives and expects to avoid the risk of endocrine disrupting surfactants (EDSs), the study tested the hypothesized roles of specific knowledge, general knowledge, and perceived uncertainty using structural equation modelling. The findings revealed that different types of knowledge played certain roles in explaining risk perception, risk acceptability, and self-protective response, which are distinguished by experience amongst the public. Evidence of the mediating role that perceived uncertainty may play in the decrease of risk perception and the increase of risk unacceptance has been provided. The insights gained from the study may help answer why the public are in favor of taking non-diet-related self-protective measures rather than changing their dietary habits, which illustrates a comparison with the basis of health belief model. The needs for building cognitive capacity amongst the public, particularly pregnant women and young mothers, and risk communication concerning endocrine disrupting contamination linked to reproductive health are highlighted.

## 1. Introduction

The endocrine disrupting surfactants nonylphenol (NP) and its ethoxylates (NPEOs) are widely used in numerous industries such as cleansing, textile, personal care product, pesticide, paper, and plastic, and end up in the environment via municipal and industrial discharge [1]. As a final metabolite in the environment, NP has raised concern due to its endocrine disrupting effects on the reproductive, immune, and central nervous systems of wildlife and humans during the past two decades [2,3,4,5,6,7,8,9]. Hormone-driven-characterized stages such as utero, infancy, childhood, puberty, and menopause are the most sensitive exposure periods [10,11,12,13,14]. Regarding carcinogenesis, recent studies have showed the relation of exposure to NP to the risk of breast cancer in women [15] and prostate cancer in men [16,17].

Acknowledging the scientific uncertainty on the causal-effect concerning NP exposure in humans, regulations on chemical safety, restriction of marketing and use, environmental quality standard, and residue control have been promulgated with the aim of ecological and health protection within the European Union. Asian countries such as Taiwan, Korea, and Japan have also launched monitoring programs as well as proposed limiting NP in textile articles, especially for children. Nevertheless, although the concentration of NP in Vietnamese urban waters has reached alarming levels in recent years [18,19,20,21], use of this chemical is still allowed and the response to ecological and health risk is negligible. This raises questions of how the society perceives the risk of these endocrine disrupting surfactants (risk perception), and how people expect to avoid the risk (risk acceptability and risk response).

Answering these questions is practically vital for designing strategic response to the risk of endocrine disrupting compounds (EDCs) in Vietnam. Taking into account the multiple applications of these endocrine disrupting surfactants (EDSs), this study focuses on their use in cleansing products, detergents, and cosmetics. The aim of the study is to examine the roles of the awareness of different levels and perceived uncertainty on EDSs risk perception, acceptability, and self-protective response among public using structural equation modeling (SEM). Hypotheses of this research are premised by the theories presented in the next section.

## 2. Materials and Methods

### 2.1. Hypothesis Formulation

In risk assessment process, understanding risk perception plays a key role in explaining risk acceptability and predicting risk response [22]. How risk is perceived could be well explained by individual capacities such as knowledge [23,24,25], or insight knowledge [26], prior personal experience [27], and self-controllability [28]. Risk perception terminology captures one’s belief in negative consequences of an event as true (risk belief) and one’s concern about the consequences (risk concern) [29].

Theories of risk perception are initially developed on the basis of value-belief-norm (VBN) theory [30,31] which intends to explain pro-environmental behavior. In expanded literature, the relationship of risk perception—risk acceptability predicting environmental action has been demonstrated by numerous scholars in the fields such as nuclear energy [32,33,34], nuclear waste [35], flood risk due to climate change [36], environmental hazard [27], and ecological risk [37]. In this study, we alternatively put our interest on people’s self-protective response to health risk.

In order to make judgments on the risk of EDSs, people are expected to have specific knowledge about those chemicals such as their utilities, impacts, and pathways of exposure in addition to a general awareness of the surrounding environment and public health situation. As suggested by Maxim, et al. [38], although people have rather good knowledge on a particular issue, they may feel uncertain or may have a perception of uncertainty [39,40] about that issue because they are not able to link the knowledge with the surrounding situation. However, the roles of specific knowledge compared with general awareness and perceived uncertainty regarding EDSs have not been well understood and proved by qualitative research. For these reasons, we were motivated to qualitatively investigate three influences: public’s cognition in terms of *general awareness* of water pollution and reproductive health problems (RHPs) and *specific awareness* of EDSs risk, as well as their *perceived uncertainty*.

**Figure 1:**
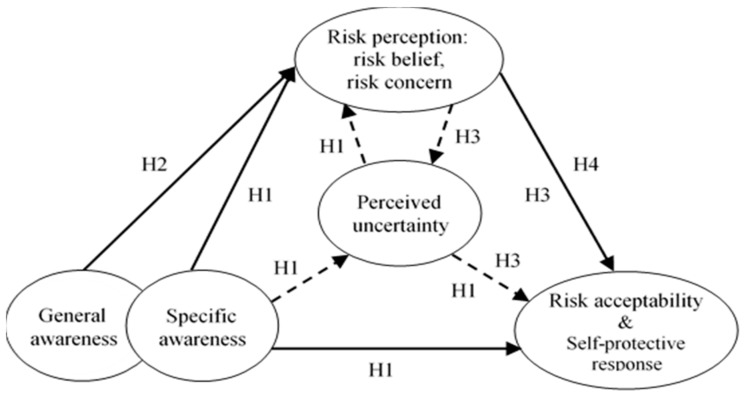
Hypothesized concept model.

The study is inspired from a publication on the health belief model [41] which provides a useful framework to explain public health behavior through four factors: perception of susceptibility, perception of severity, perception of barriers, and perception of benefits. It is suggested that perception of uncertainty is one of the barriers in decision-making [42], thus it could be a barrier against behavioral changes. From that logic, we hypothesized that perceived uncertainty might play a mediating role in the relationship of knowledge of EDSs with risk perception (in terms of risk belief and risk concern), as well as in the relationships of risk perception with risk acceptability and resulting self-protective response. Four hypotheses were formulated as follows (Figure 1):

**Hypothesis** **1** **(H1).** Perceived uncertainty mediates the positive relationships of specific awareness of EDSs with risk belief, risk concern, risk acceptability, and self-protective response.

**Hypothesis** **2** **(H2).** General awareness of water pollution and RHPs also has direct and positive effects on risk belief and risk concern.

**Hypothesis** **3** **(H3).** Perceived uncertainty plays a mediating role in the positive relationship of risk belief and risk concern with risk acceptability and self-protective response.

**Hypothesis** **4** **(H4).** People distinguished by experience and status of pregnancy and child differ in the relationships of risk perception with risk acceptability and self-protective response.

### 2.2. Research Design

Data for this research was obtained randomly from 331 ≥18-year-old participants of all genders, including pregnant women and young mothers. The main study area is Hochiminh city, which is the biggest urban center in Vietnam. Therefore, most of the participants who accounted for 92% of the sample were recruited across social structure in sub-urban and urban Hochiminh city (23/24 districts). The remaining participants were doctors and city-level governmental officers from Danang city, another important urban center of Vietnam. Before the official data collection, close-ended questionnaires were pretested with 50 people to make sure all questions were clear and understandable. As a result, we added a basic definition of EDSs to help participants relate the questions about the chemicals to the information they have read or learnt. Participants were met at their homes in residential areas and worker houses, universities, research institutions, industrial and governmental agencies. Most of pregnant women and health care providers were accessed at hospitals. The questionnaires were self-administered.

### 2.3. Measures

#### 2.3.1. Socio-Demographic Variables

Socio-demographic measures included age, gender, residence location, level of education, working field, marriage status, pregnancy and child status. An interval scale for age (18–25/26–40/41–60/>60) and a nominal scale for gender (female/male) were used. The residence location of participants was recorded as one of 24 districts of Hochiminh city or Danang city. Participants selected their education level from a nominal scale of junior high school/high school/undergraduate/graduate/postgraduate/others. Working field included housewife/worker/student/household business/education or research (to be further specified by fields, such as biology, chemistry, environmental science)/medical and health care (e.g., doctor, health care technician, nurse)/industrial manufacture/service/government (to be specified into sectors)/non-governmental organization/social organization/other (self-indicated). Marriage status (single/married), pregnancy status (pregnant/non-pregnant/tentatively pregnant), and child status (no child/having at least one child: youngest child age of <6/having at least one child: youngest child age of ≥6) were also recorded.

#### 2.3.2. Experienced versus Lay Public

From the socio-demographic information, we defined experienced public based on their working fields. Accordingly, the experienced public were those working in the environmental governmental sector, research and education institutions (e.g., biological, chemical, environmental researchers, and lecturers), and medical and health care sector (e.g., doctors and health care technicians). We assumed that they had information about the environmental situation, and biological-chemical-medical background that might be relevant to our research topic. Lay public were the remaining participants.

#### 2.3.3. Pregnant Women and Young Mothers versus the Remaining Respondents

We distinguished pregnant women and young mothers using information on pregnancy and child status. They included women who were in pregnancy, those having at least one child at the age of less than 6 years old, and those planning to have a child.

#### 2.3.4. General Awareness of Water Pollution and Reproductive Health Problems

This construct was derived from eight questions where the respondents selected their answers based on a five rating-point Likert scale from “totally disagree” to “totally agree”. The first two questions asked about people’s awareness of the intrinsic value of the nature, and whether it should be protected unconditionally. The second set of questions was to explore public’s awareness of urban river and canal pollution, and whether that situation might adversely affect the health of human beings. Questions 5 and 6 aimed to investigate the publics’ knowledge of some water pollutants that might cause RHPs such as pesticides, dioxins, bisphenol A, phthalates, heavy metals, and whether they knew about the increasing RHPs these days. The last two questions were relevant to pathways of exposure as food and drinking water produced from contaminated water sources that might cause RHPs. 

#### 2.3.5. Specific Awareness of Endocrine Disrupting Surfactants

The construct of specific awareness of EDSs was derived by six questions. The first question asked about public’s cognition of the urban water contamination by EDSs. The next five questions explored public’s knowledge in more details: EDSs and their sources, negative impacts as feminization in wildlife (fish) and reproductive disorders in humans, pathways of exposure via consuming riverine fish and drinking water. The generation of those questions was based on the knowledge of EDSs contamination [18,19,43], the sources of EDSs [1], and the effects on wildlife and humans [44]. This construct adopted a mixed scale that measured the correctness of the participants’ answers. The respondents were asked to make their judgments about the given information via seven options: “do not know”, “wrong”, “low certain”, “moderately uncertain”, “neutral (unsure)”, “moderately certain”, and “highly certain”. We supposed that people selected “do not know” because they had never heard or learnt about EDSs. Those who judged the statements as “wrong” might have known about EDSs but their knowledge might not be correct. Others who had certain knowledge of EDSs might select from “low certain” to “highly certain” depending on their confidence.

#### 2.3.6. Risk Perception

Public risk perception was examined through their belief (two questions) and concern about EDSs (two questions). The former questions asked about people’s belief that EDSs in detergents, cleansing agents, and cosmetics might cause disorders of reproductive system in fish (e.g., feminization of fish) and disorders of reproductive system in humans. The latter investigated people’s concern about the chemicals and the effects on humans, taking into account their self-controllabilities as not to consume riverine fish and to control from exposure to the chemicals. Respondents could select the best answers from a five rating-point Likert scale (“totally disagree”–“totally agree”).

#### 2.3.7. Perceived Uncertainty

We supposed that the public might feel uncertain about the impact of EDSs for some reasons. Therefore, in the corresponding questions, we linked the uncertainty about the adverse effects on fish and humans with three reasons: information about these surfactants has not appeared widely in media, insufficient attention and warning from scientists, and disorders in reproduction could be due to other reasons. This construct was measured by a five rating-point Likert scale (“totally disagree”–“totally agree”). 

#### 2.3.8. Risk Acceptability

Risk can be acceptable after it has been reduced further to be negligible [45]. Being aware that setting the level of acceptable risk was difficult and sensitive [46], we adopted indirect questions about what the government should do regarding mitigation or/and prevention of EDSs risk. We were interested in the implied levels of controlling the concentration of EDSs from detergents, cleansing agents, and cosmetics in rivers. The first level was for the sake of aquatic life in general and riverine fish in particular (nature-oriented solution), whereas the second was for the human health and wellbeing (human-oriented solution). The third level aimed at a more prevention-oriented solution that required a control, giving that the adverse effects on human health were uncertain. A five rating-point Likert scale from “totally disagree” to “totally agree” was also adopted in this measurement.

#### 2.3.9. Self-Protective Response

We supposed that if people believed that they were exposed to EDSs via food and drink, they might take preventive measures to protect themselves. In this case, we suggested four options: to have frequent health checkup, to have a drinking water checkup, to install a drinking water filter, and to consume riverine fish less regularly. A similar five rating-point Likert scale as aforementioned was used for this measurement.

### 2.4. Analysis Methods

We tested the hypotheses using structural equation modeling (SEM) by AMOS version 23 (IBM Inc., Chicago, IL, USA). This method was based on multiple regression equations which allowed researchers to examine complex relationships among latent constructs [47]. One of the advantages of SEM was that the outcome validation could be enhanced thank to the reliability of the constructs [48]. To do that, we firstly conducted an exploratory factor analysis (EFA) to check the appropriation of the proposed scales. The process was followed by a confirmatory factor analysis (CFA). CFA served as an intermediary but essential step to provide supports to the SEM validation. After all, the proposed hypotheses were tested using SEM. The full procedures for SEM applied in this study were recommended by Gaskin [49].

#### 2.4.1. Exploratory Factor Analysis Procedures

**Figure 2:**
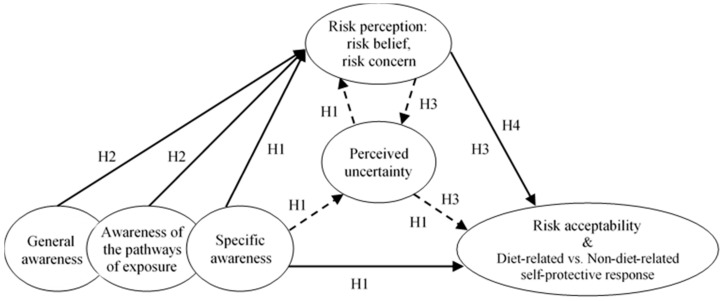
Modified hypothesized concept model.

The sample size was 331 and missing values of each variable of maximum 2% were replaced by median values for ordinal variables. We firstly conducted an EFA for 27 items using maximum likelihood extraction method and subjected to a varimax rotation. This factor analysis suggested a seven-factor solution with the accumulative extraction sum of squared loadings of 61.2%. Kaiser-Meyer-Olkin (KMO) value of the test was 0.84, which indicated that the observed correlation matrices were factorable. The KMO measure of sampling adequacy of greater than 0.5 is acceptable to proceed a factor analysis [50]. Based on the loadings of items, the following modifications were made:

- The scale “general awareness of water pollution and RHPs” was split into two, which were named as “general awareness of water pollution and RHPs” and “awareness of the pathways of exposure that might affect reproductive health”, comprising of six and two items, respectively (Figure 2). Since the items of the latter scale may require more insight knowledge compared with those in the former scale, this modification is acceptable.
- “Risk belief” items of the “risk perception” scale showed an unclear trend of loading on two factors. Therefore, we decided to treat the “risk belief” items as components of a separate factor in the following CFA.
- The first three items of the “self-protective response” scale showed rather good loadings on one factor. Since these questions were irrelevant to food, we named this factor as “non-diet-related self-protective response”. The last item of this scale particularly relevant to a change in daily riverine fish diet presented a loading on none of the factors, so we treated it as a “stand-alone” endogenous variable in CFA, namely as “diet-related self-protective response”.

Items of the remaining scales showed good loadings. All factor loadings (standardized regression weights) were statistically significant (*p* < 0.001). Next, based on the EFA results and substantive interpretation, internal consistency of each scale was examined using Cronbach’s alpha. All scales showed high Cronbach’s alphas of from 0.79 to 0.91, which indicated good internal consistency. The alpha coefficients of 0.70 and above indicate sufficient reliability “in the early stages of research on predictor tests or hypothesized measures of a construct” [51]. The results of EFA were summarized in Table 1.

It was suggested from the EFA that awareness was composed of three levels (three factors), risk perception including risk belief and risk concern (two factors), and self-protective response including “non-diet-related self-protective response” (a factor) and “diet-related self-protective response” (a “stand-alone” endogenous variable). The modification of the hypothesized model was illustrated in Figure 2.

#### 2.4.2. Confirmatory Factor Analysis Procedures

We adopted the CFA procedures proposed by Gaskin [49]. This process included four main steps: (1) model fit; (2) examining the validity and reliability of the factors in CFA test; (3) checking common method bias, and (4) testing measurement model invariance across groups. Maximum likelihood estimation method was used because it could work with light to moderate skewness and kurtosis with rather small sample size (100–400 subjects) [52]. Goodness of model fit was assessed based on Chi-square/*df* ratio (CMIN/*df*), Comparative Fit Index (CFI), Tucker Lewis Index (TLI), Standardized Root Mean Square Residual (SRMR), Root Mean Square Error of Approximation (RMSEA), and *p* of Close Fit (PCLOSE). The CFI and TLI values of greater than 0.90 reflect an acceptable fit to good fit (≥0.95) [53,54]. RMSEA values of from 0.05 to 0.08 indicate an acceptable fit and those of below 0.05 represent a good fit, whereas SRMR values of less than 0.08 generally show a good fit [54]. The PCLOSE tests the null hypothesis that the RMSEA is of 0.05, therefore PCLOSE values of greater than 0.05 indicate close-fitting models [55]. The results of CFA were summarized in Table 2 and Supplementary Materials: Tables S1 and S2.

The initial measurement model achieved good model fit with *χ*^2^ = 675.0, *df* = 293; CMIN/*df* = 2.3, CFI = 0.92, TLI = 0.91, SRMR = 0.06, and RMSEA = 0.06, whereas the PCLOSE test was statistical significant (*p* < 0.01), which suggested that the initial model was not so close-fitting (Table 2). We accepted modification indices that suggested covariances in three pairs of observed variables: GA5 and GA6 were both about general awareness of RHPs, SA1 and SA2 both relevant to knowledge of EDSs sources, and SA5 and SA6 investigating knowledge of pathways of exposure to EDSs.

Construct reliability and validity were assessed using criteria suggested by Hair [56]. The latent constructs achieved a composite reliability (CR) since all CR values exceeded 0.6 (CV = 0.8–0.9). Most of the average variance extracted (AVE) values were greater than 0.5 except for the construct of specific awareness of EDSs (AVE = 0.5), indicating an acceptable reliability of the measurement model in measuring the constructs. The maximum shared variance (MSV) values and the average shared variance (ASV) values of the constructs were smaller than the AVE values, and the square root of the AVE values were greater than the inter-construct correlation coefficients. Therefore, the discriminant validity for all constructs was also achieved. Details of the construct reliability and validity, and the construct discriminant validity were provided in the Supplementary Materials (Tables S1 and S2, respectively).

In the next step, we aimed to check common method bias using “controlling for the effects of a single unmeasured latent method factor” [57]. A first-order common latent factor (CLF) was added into the measurement model. A chi-square test for the difference between the CLF unconstrained model and the CLF full-constrained model was conducted. The resulting chi-square difference was statistically significant (Δ*χ*^2^ = 147.9, Δ*df* = 27, *p* < 0.01). Hence, we had significant shared variance that led us to retain the CLF in the baseline model. The unconstrained model where the variance of CLF was set to 1 had improved fitting indices: *χ*^2^ = 527.1, *df* = 266; CMIN/*df* of 2.0, CFI = 0.95, TLI = 0.93, SRMR = 0.05, RMSEA = 0.05, and PCLOSE = 0.135 (Table 2). Factor scores that accounted for the shared variances of the CLF were imputed from CLF unconstrained model.

We further tested configural invariances for the measurement model controlled by two grouping variables: experience and pregnancy and child status, and we obtained an adequate goodness of fit (Table 2). Metric invariance test for the measurement model controlled by pregnancy and child status revealed that forcing two groups together was substantially different than letting them be estimated freely (Δ*χ*^2^ = 37.3, Δ*df* = 19, *p* < 0.01). Whereas, the metric invariance test for the measurement model controlled by experience resulted in a non-significant chi-square difference (Δ*χ*^2^ = 19.3, Δ*df* = 19, *p* = 0.438), indicating that the groups were non-significantly different at measurement model level; however, they could be different at path level. Therefore, it was suggested to check structural weights model invariance and path differences between groups.

#### 2.4.3. Structural Equation Modelling Procedures

Before conducting SEM, multivariate assumptions including influential, multicollinearity, and normality by groups were examined (see Supplementary Materials: Tables S3 and S4). Using Cook’ s distance, we decided to remove three records (Cook’s distance of from 0.13 to 0.21) that deviated much far from others in order to eliminate their influences on the results. The new sample size was 328 (331 − 3 = 328). The multicollinearity test resulted in the tolerance values of 0.60–0.86 and the VIF values of 1.2–1.7, which were satisfactory (tolerance > 0.1 and VIF < 3). A Shapiro-Wilk normality test (*p* < 0.01) was performed on eight factors and one dependent variable showed a statistically significant non-normality of the data set. A visual inspection of their histograms, normal Q-Q plots and box plots across groups revealed light to moderate deviations from normal distributions. The *z*-values of the skewness for the experienced public, the lay public, the pregnant women and young mothers, and the remaining population were in the ranges of −5.6–1.6, −8.8–3.8, −8.2–3.2, and −8.8–2.4, respectively, and the *z*-values of the kurtosis were −1.8–3.7, −3.4–5.6, −2.7–8.2, and −3.3–5.2, respectively. As an exception, “general awareness of water pollution and RHPs” construct had extreme *z*-values of skewness from −8.8 to −15.0 and *z*-values of kurtosis from 11.0 to 27.8. 

Imputed factor scores from CLF unconstrained measurement model and maximum likelihood method of estimation were used in our path model. After accepting the covariances between GA and AP, and between e3 and e4 (the residuals of “risk acceptability” and “non-diet-related self-protective response”, respectively, the baseline path model had significantly improved in goodness of fit with *χ*^2^ = 11.6, *df* = 8; *p* = 0.172, CMIN/*df* = 1.4, CFI = 0.99, TLI = 0.98, SRMR = 0.03, RMSEA = 0.04, and PCLOSE = 0.636 (see Table 2). The shared variances were understandable because the former pair of constructs might be relevant to a more global concept of environmental pollution and public health, whereas the latter pair of constructs might be affected by a certain factor. Afterwards, we started to test the hypothesized relationships (H1, H2, and H3). The level of confidence of the hypothesis tests was set at 95% (α = 0.05). Testing of hypothesis H4 was conducted in two stages. We first made group comparisons (multi-sample approach) suggested by Reinecke [58] in order to obtain an insight of the structural weights invariances using the same baseline model. The invariance test revealed a significant difference between the experienced and the lay public at structural weights model level (Δ*χ*^2^ = 68.1, Δ*df* = 26; *p* < 0.01), whereas the model controlled by pregnancy and child status showed a non-significant difference between the groups (Δ*χ*^2^ = 25.3, Δ*df* = 26; *p* = 0.501). Since those invariance tests were still at model level, we further examined the parameter estimates invariances in single relationships of risk perception with risk acceptability and with self-protective response between groups.

## 3. Results

### 3.1. Participants

The sample was representative by early adults (26–40: 54.4%), young adults (18–25: 37.9%), middle adults (41–60: 6.7%), and latter adults (>60: 0.9%). Female and male rates were 64.4% and 35.6%, respectively. Most of the participants achieved undergraduate level of education, a rate of 73.5%, whereas the shares of junior high school, high school, graduate, postgraduate, and others were 2.8%, 12.3%, 7.7%, 0.6%, and 3.1%, respectively. Among the participants living in Hochiminh city, who accounted for 92.2% of the sample, 67.4% were from urban and 24.8% were from suburban districts. Only 7.8% of the participants were from Danang city. Based on the aforementioned criteria of experience, 29% of the participants were categorized as experienced public, and 71% were defined as lay public. The share of pregnant women and young mothers was 36%, and the remaining group accounted for 64% of the sample. The numbers of missing value were one for age, two for gender, four for education, and six for residence location in the total of 328 records.

### 3.2. Mean Values and Bivariate Correlations

The minimum, maximum, and mean values of each construct and the correlation coefficients between the constructs were presented in Table 3 and Table 4, respectively. The results in Table 3 revealed a high levels of general awareness of water pollution and RHPs (GA: µ = 5.2–5.4), risk acceptability (RAC: µ = 5.3–5.6), and non-diet-related self-protective response (NDSP: µ = 5.2–5.5) amongst the public. The levels of awareness of the pathways of exposure that may affect reproductive health (AP: µ = 3.7–3.8), risk belief (RB: µ = 3.8–4.1), and risk concern (RC: µ = 4.0–4.1) were moderately high. Whereas, the public had rather low level of specific awareness of EDSs (SA: µ = 1.7–1.8) with low uncertainty (UN: µ = 1.9–2.1), and they showed neutral tendency of diet-related self-protective response (DSP: µ = 2.7–3.0).

Because SEM was based on Pearson correlation [47] whereas the data set showed deviations from normality across constructs, both Pearson correlation (r) and Spearman’s rho (r_s_) were examined. The parametric and non-parametric estimations resulted in comparative bivariate correlations in terms of magnitude and statistical significance (see Table 4). The results in Table 4 revealed strong correlations of RAC with GA (r = 0.427, r_s_ = 0.454, *p* < 0.01) and RB (r = 0.478, r_s_ = 0.362, *p* < 0.01), and a weaker relationship with RC (r = 0.338, r_s_ = 0.330, *p* < 0.01). Meanwhile, the correlations of RAC with AP and SA were statistically non-significant. NDSP was positively and significantly related to three levels of awareness and risk perception where the relationship with RB was strongest (r = 0.472, r_s_ = 0.486, *p* < 0.01). However, DSP showed non-significant relationships with the constructs of awareness and risk perception. Whilst UN was negatively associated with NDSP (r = −0.210, r_s_ = −0.252, *p* < 0.01), it was positively correlated with DSP (r = 0.200, r_s_ = 0.211, *p* < 0.01).

### 3.3. Hypothesis 1

**Figure 3:**
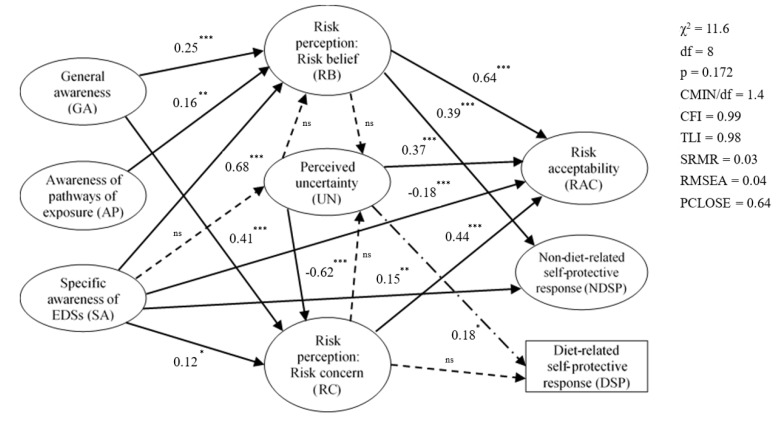
Final model of the determinants of risk perception, risk acceptability, and self-protective response. Notes: Direct effects: thick solid arrows; moderating effects: slim solid and dot lines; indirect effects: combinations of a slim dot line (SA/RB to UN) and a long dash dot line (UN to DSP). * *p* < 0.05; ** *p* < 0.01; *** *p* < 0.001; ^ns^ non-significant.

The outcome model and the results from the path analysis were summarized in Figure 3 and Table 5, respectively. The model was composed of eight constructs and one observed endogenous variable. Only statistically significant standardized regression weights (β) were showed, whereas paths without beta indicated non-significant effects.

The findings revealed that the effects of specific awareness of EDSs (SA) on risk belief (RB) and risk concern (RC) completely supported H1. Accordingly, the paths from SA to RB (β = 0.68, *z* = 4.83), and to RC (β = 0.12, *z* = 1.62) were positive and statistically significant. SA appeared to have a strong association with RB, suggesting that the insight of EDSs was a determinant of perceiving EDSs risk. In addition, this relationship was weakened in the presence of an uncertain feeling about EDSs risk, indicating the mediating role of perceived uncertainty (UN). Indeed, when the relationship was separately investigated, the direct effects without- and with-mediation were 0.54 (*p* < 0.01) and 0.48 (*p* < 0.01), respectively. Indirect effects of SA on RB through UN were non-significant, thus they were not showed in Figure 3.

Nonetheless, these results seemed not similar for RC. SA slightly associated with RC with a partial mediation through UN. Among the indirect relationships, only the path from UN to RC was significant (β = −0.62, *z* = −3.33). The negative beta implied that the less uncertainty about EDSs risk, the more concerning about consequences people might feel.

The direct association of SA with risk acceptability (RAC) was marginally negative and significant (β = −0.18, *z* = −3.62). It was partially mediated by UN, which exerted a positive and significant influence on RAC (β = 0.37, *z* = 7.70). These inverse trends could be understood in a way that people who were more ambiguous about EDSs risk showed more in favor of supporting EDSs controlling strategies or the unlikeliness of risk acceptance. This partially supported H1.

Whist SA successfully explained non-diet-related self-protective response (NDSP) with a positive and direct effect of 0.15 (*z* = 2.60, *p* < 0.01), it indirectly influenced diet-related self-protective response (DSP) through UN. Among the indirect paths, only the path from UN to DSP was significant (β = 0.18, *z* = 2.99, *p* < 0.01), suggesting that those who felt more uncertain about EDSs risk showed more unlikely to consume riverine fish. UN exerted no mediating role in these relationships, which did not support H1.

### 3.4. Hypothesis 2

It was revealed that the paths from general awareness of water pollution and RHPs (GA), and awareness of the pathways of exposure that may affect reproductive health (AP) to RB (β = 0.25, *z* = 5.19 and β = 0.16, *z* = 2.95, respectively), and the path from GA to RC (β = 0.41, *z* = 6.35) were direct and positive, which supported H2. Whereas, the path from AP to RC was non-significant, which failed to support H2. We did a post-hoc analysis for this unsupported direct effect and we did have power to detect the non-significant direct effect of AP on RC that might be existing. Therefore, we were confident that this non-significant effect was truly non-significant with the power to detect of 100% with 5% confidence level. The results of H2 are provided in Figure 3 and Table 5.

### 3.5. Hypothesis 3

Perceived uncertainty (UN) played a partially mediating role in enhancing the relationships of RB and RC with RAC. The effects without- and with- mediation were 0.45 (*p* < 0.01) and 0.56 (*p* < 0.01) for RB, and 0.28 (*p* < 0.01) and 0.44 (*p* < 0.01) for RC, respectively, suggesting that people were more in favor of preventive strategies when they were unsure about EDSs risk. Total effects of RB and RC on RAC were 0.64 (*z* = 12.91, *p* < 0.01) and 0.44 (*z* = 9.84, *p* < 0.01), respectively. UN exerted a full mediation on the path from RC to DSP although the total effect of this relationship was non-significant. These findings, to some extent, provided supporting evidence to H3. 

RB successfully explained NDSP with a positive and direct effect of 0.39 (*z* = 6.81, *p* < 0.01), whereas RC presented a non-significant effect on this type of tentative behavior. The relationship of RB with DSP was indirect through UN, where only the indirect path from UN to DSP was significant. UN exerted no mediating role in these relationships, which did not support H3. Within our path model, variance explained for RB, RC, RAC, NDSP, and DSP were 0.24, 0.25, 0.46, 0.24, and 0.04, respectively. The results of H3 are summarized in Figure 3 and Table 5.

### 3.6. Hypothesis 4

#### 3.6.1. Experience Difference

As the results of the chi-square tests, the differences in the paths from risk belief to risk acceptability (Δ*χ*^2^ = 5.0, Δ*df* = 1; *p* = 0.025) and from risk belief to NDSP (Δ*χ*^2^ = 4.6, Δ*df* = 1; *p* = 0.031) were significant between the experienced and the lay public, which supported H4 (see Table 5). The effects of risk belief on risk acceptability and on NDSP were slightly stronger for the experienced public (β = 0.67 and β = 0.55) than the lay public (β = 0.53 and β = 0.29), respectively.

#### 3.6.2. Pregnancy and Child Status Difference

The chi-square tests revealed a non-significant difference in the hypothesized paths between pregnant women-young mothers and the remaining respondents (see Table 5).

## 4. Discussion

### 4.1. Difference between the Experienced and the Lay Public Looking from the Knowledge Aspect

**Figure 4:**
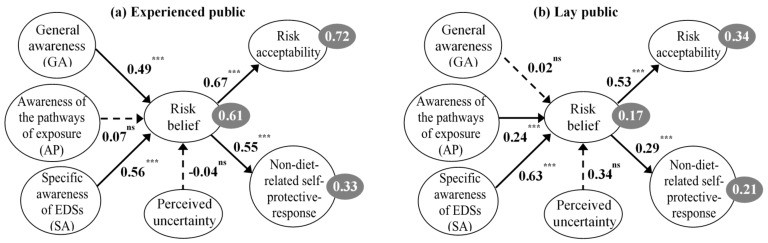
Contribution of three levels of knowledge on the risk belief of the experienced public (**a**) and the lay public (**b**). *** *p* < 0.001; ^ns^ non-significant.

The findings supporting H4 revealed that the patterns of risk belief and their relation to NDSP and risk acceptability differed between the experienced and the lay public. It is argued that lay public tend to make judgments on health and environmental risk from their cognitive heuristics [59,60], whereas experts draw conclusion based on knowledge with assumptions and assessment techniques [61]. Compared with those experts participating in risk studies that have been reported, the experienced sample in our research is much more diverse in expertise, however they do share some characteristics in common such as bio-chemical knowledge of life science and/or scientific information on water pollutants and impacts, which distinguish them from the lay public. Therefore, it would be interesting to inspect the difference in the patterns of risk belief between the experienced and the lay public from the knowledge aspect (described in Figure 4). 

The multi-sample structural weights model showed that risk belief was attributed to GA much more for the experienced respondents (β = 0.49, *p* < 0.01) than for the opponents (β = 0.02, non-significant), respectively. Experienced people are supposed to have insights of the urban environmental pollution from domestic and industrial activities, which are claimed responsible for EDCs discharge [1,18,19,62]. In an inverse trend, the lay public related AP to EDSs risk more than the opponents did, with corresponding β = 0.24 (*p* < 0.01) and β = 0.07 (non-significant). Surprisingly, SA contributed comparatively to the risk belief of both experienced and lay public with β = 0.56 (*p* < 0.01) and β = 0.63 (*p* < 0.01), respectively. The results seem illogical if we fail to consider the perceived uncertainty. Indeed, it was found that uncertainty exerted a larger effect on risk belief for the lay public (β = 0.34) than for the opponents (β = −0.04) although those direct effects were non-significant. This implies that experts’ perceived risk is influenced by knowledge, whereas lay public are probably influenced by their cognitive heuristics. Our finding that three levels of knowledge benefit the experienced and the lay public in different ways deepens the existing theories in an effort to explain ecological and health risk perception in modern society. 

The model appeared more parsimonious for the experienced sample to explain risk belief, risk acceptability, and NDSP with variance explained of 0.61, 0.72, and 0.33 than for the inexperienced sample with variance explained of 0.17, 0.34, and 0.21, respectively. Therefore, it is supposed that there are other determinants that could explain EDSs risk belief better for lay public, such as education [63,64], income [65], and media [66]. We also consider about the inequality in sample sizes with 233 inexperienced respondents and 95 experienced respondents.

### 4.2. Perceived Uncertainty and Cognitive Bias

In this study, although specific awareness of EDSs (SA) exerted a significant effect on people’s risk belief, this effect was weakened by the uncertain feeling. In addition, the respondents showed less concern about the EDSs risk in the presence of uncertainty. The results, on the one hand, are in agreement with scholars in the field of public health who highlight the role of knowledge in explaining risk perception [23,24,25]. Noticeably, although the respondents seemed to have moderately low awareness of the EDSs in terms of contamination situation, sources, impacts and pathways of exposure, they showed rather high belief of the chemicals’ impacts and concern about this issue. Therefore, it is suggested that the public may have cognitive bias that probably affects their perceived risk [67,68]. In this situation, cognitive bias should be understood as “a skewing from a standard or reference point”, where the following uncertainties may be relevant: “availability—how humans account for rare events depends upon whether they have experienced them or not”, and “anchoring—humans cannot move from preconceptions, but instead anchor to them even in light of new data/information” [69]. Our introduction about the research as well as the questionnaire itself may stimulate the public to relate EDSs risk with similar preconceptions. This may bring another aspect into the discussion of Pligt [70] and Maxim, Mansier and Grabar [38] that possible bias in health risk estimation is dependent on risk communication context, risk characterization, as well as personal and cultural characteristics. 

On the other hand, the finding that perceived uncertainty due to insufficient risk communication and scientific uncertainty of EDSs impact leads to a decrease in risk perception provides supporting evidence to Funtowicz and Ravetz [71] and Patt and Schrag [72]. The result is closely connected to the finding that perception of uncertainty is attributed to an inability to precisely identify the causal relationship (e.g., male reproductive disorders due to exposure to EDSs) as well as questions about data, methodology, extrapolation and epistemological validation [38]. 

### 4.3. Difference in How People Take Non-Diet-Related and Diet-Related Self-Protective Response

The direct and positive effect of risk belief on NDSP is in line with the findings of Lindell and Hwang [27], Botzen, et al. [73], Siegrist and Gutscher [74], and Miceli, et al. [75] in environmental hazard domain, and of Sjöberg [76], Mak and Lai [77], and Remoundou, et al. [78] in the safety and health protection domain. In spite of that, some studies indicate a minor role of risk perception on precautionary solution [79]. It could be suggested that people are in favor of taking the NDSP as preventive solutions regardless their ambiguous knowledge of EDSs, feeling of uncertainty, and concern about the impacts. In consistency, Heath and Tversky [80] have found that people with unconscious information tend to show more risk-averse attitudes, and previous works of Vaughan [81] and Colvin, et al. [82] have provided evidence that self-protective actions are likely taken by those who believe that precautious solutions are helpful.

In contrast, perceived uncertainty was mainly responsible for being unlikely to take DSP, as consuming less riverine fish, despite of high EDSs risk perception. The possibility is that people perceive uncertainty as a barrier and/or they perceive more benefits than reducing exposure. Indeed, scholars have provided evidence that perception of uncertainty is among barriers in decision making, where the uncertainty is related with knowledge [42], experience [83], and consequence of a decision [84,85,86]. In addition, people are likely to prioritize the advantageous sides of products (e.g., overall nutrition of fresh food) whilst they perceive deviating from common diet habits in order to mitigate exposure to EDCs as a barrier [26]. Noticeably, the crisis of massive fish deaths along the sea coast of Vietnam that occurred just four months before this survey was conducted significantly limited marine fish supply as well as driving people from consuming seafood to riverine fish. This socio-environmental factor may contribute to explain why people are unlikely to consume less riverine fish. 

### 4.4. Contribution to the Health Belief Model

The previous discussions have elucidated the role of specific awareness in explaining risk belief and risk concern, which could be referred to the perceived susceptibility that is described in the health belief model (HBM) [41]. 

**Figure 5:**
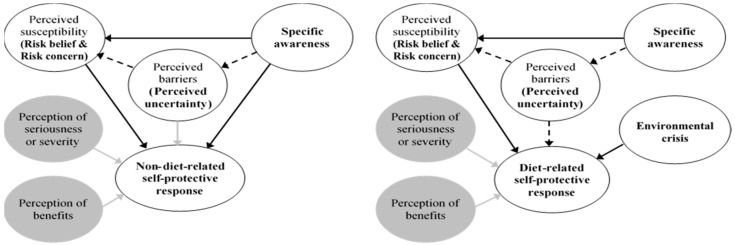
The role of specific awareness and perceived uncertainty in contributing to the HBM [41]. Note: Shaded boxes and gray lines: the constructs and the relationships described in the HBM; unshaded boxes and black lines: the modifications by this study; solid lines: direct effects; dot lines: indirect/moderation effects.

In addition, the findings have provided supporting evidence to the mediating role of perceived uncertainty in explaining risk belief, risk concern, and diet-related self-protective response. Perceived uncertainty is referred to the perceived barrier that lays the theoretical foundation for our hypotheses, whilst the tendency of taking self-protective response directly determines one’s behavior [87], both of which are the HBM components. The theoretical contribution to the HBM is illustrated in Figure 5.

### 4.5. Explained Variances and Potential Influencing Factors

Within the path model, 24% and 25% of variances of risk belief and risk concern were explained, respectively. Perceiving risk consequences, in reality, is affected by various outrage factors, among which are familiarity, fairness, and dread [88]. It follows a pattern that higher perceived risk would be likely with outrages. In this study, the familiarity could be related to an acquaintance with or an experience of RHPs such as abnormalities in reproductive organs, feminization in males, whereas fairness implies an inequality in risk suffering by age and gender [44]. Regarding dread, cancers are probably more dreaded than endocrine system malfunction. The three outrages could be potential factors to explain EDSs risk perception. Variance explained for risk acceptability, NDSP, and DSP were 0.46, 0.24, and 0.04, respectively. High responsiveness is related to other factors such as income [27], belief in the effectiveness of the measure [81,82], and perceiving benefits [26,41]. Within the scope of this study, the roles of those factors in behavioral decision have not been demonstrated.

### 4.6. Implication for Risk Communication Practice and Decision-Making Process

Communication of EDSs risk would benefit from acknowledging the public’s cognitive bias and perception of uncertainty with regards to the specific awareness of EDSs. Particularly, we highlight the importance of communicating the causal relationship between EDSs exposure and RHPs as suggested by Maxim, Mansier and Grabar [38]. The finding of non-significant differences in risk perception, risk acceptability, and self-protective response between pregnant women-young mothers and the remaining respondents, to some extent, agrees with the finding that pregnant women may not perceive the risk of food consuming behaviors on the adulthood of their babies [26]. Therefore, this is a key point for risk communication in practice as one of the medical care considerations in early preparation for pregnancy, which targets pre-pregnancy women. The study has provided original information on the public awareness of different levels, their perceived EDSs risk, risk acceptability, and tendency of self-protection with both non-diet-related and diet-related measures, which are essential for decision-making process.

### 4.7. Strength and Limitation

The strength of this study is that it is based on a validated methodology with reliable measurement scales and constructs, goodness of model fit, considerable R squared, and statistical significance of difference chi-square statistics and individual paths. It is suggested that this model could be replicated for testing other causal paths. In some instances, we have a confidence that our results could be generalized to urban population in Vietnam.

Nevertheless, this study does have limitation that a 328-respondent sample is not a large sample, so we have deviations from the normality of distributions. Although normal distribution is hardly obtained in social research, it is our responsibility to report for its possible hidden effects on our results. As aforementioned, this study has been carried out in the context of marine environmental crisis in Vietnam. Therefore, the public are supposed to be psychologically affected by this outrage factor. When testing the measurement model, we have found a significant shared variance of the CLF which reflects a common method bias. This type of CLF could be relevant to “transient mood state” effect, which “refers to the impact of relatively recent mood-inducing events to influence the manner in which respondents view themselves and the world around them” [57]. Consequently, the CLF may share a common effect of the fear on various environment-related constructs such as GA, AP, risk perception, and acceptability.

## 5. Conclusions

Our findings revealed rather high perception of EDS risk amongst the respondents, who tended to be in favor of risk mitigation strategies at the governmental administration level and more likely to take non-diet-related preventive measures. The respondents seemed consistent with their diet habits like riverine fish consumption. The patterns of risk belief and their relation to non-diet-related self-protective response and risk acceptability differed by experience amongst the public. Communicating the societal risk perception and tentative behaviors, one the one hand, we convey the public concerning attitudes to relevant stakeholders. On the other hand, the study informatively contributes to and helps enhance the legitimacy of risk decision-making process in Vietnam. As among the few studies in this field using a systematic questionnaire survey and SEM, the study elucidates the roles of three types of knowledge that distinguished by experience, and the mediating role of perceived uncertainty in explaining EDSs risk perception, acceptability, and self-protective response, which have not been mentioned by other scholars.

Two policy implications could be suggested. First, a national policy for raising awareness amongst the public with a focus on pregnant women and young mothers is needed. It should emphasize social interventions, such as health education and risk communication programs. Particularly, EDCs contamination linked to reproductive health should be a critical part in such programs. Second, since studies on the EDSs contamination are rare in Vietnam, research policy should support expanded work in this area, such as the bioaccumulation of and the exposure to these substances.

We suggest testing this model for other EDCs categories as well as extending the governing factors of risk perception to educational level, income, and media. Additionally, future research should focus on biased media coverage suggested by Slovic, Fischhoff and Lichtenstein [60] and Lindell and Perry [89,90] in order to explain perceived uncertainty. This is supposed to contribute greatly to EDCs risk communication.

## Figures and Tables

**Table 1:** Alpha coefficients and factor loadings (*n* = 331).

Construct		Variable		Standardized Regression Weight
General awareness of water pollution and reproductive health problems (GA) (α = 0.87)		GA1	I believe that the nature is valuable for its own sake.	0.643
		GA2	I believe that to protect the environment unconditionally is important.	0.829
		GA3	Rivers and canals in our cities are polluted at different levels.	0.728
		GA4	Polluted rivers may have adverse effects on the health of human beings.	0.851
		GA5	Some water pollutants that may negatively impact the reproductive health of human beings are pesticides, dioxins, bis phenol A, phthalate, heavy metals (e.g., Cd, Pb, Hg)	0.640
		GA6	More people have reproductive health problems these days.	0.726
Awareness of the pathways of exposure that may affect reproductive health (AP) (α = 0.88)		AP1	Reproductive health problems are possibly caused by food produced from polluted water.	0.931
		AP2	Reproductive health problems are possibly caused by drinking water exploited from contaminated water resources.	0.841
Specific awareness of endocrine disrupting surfactants (SA) (α = 0.85)		SA1	These days, urban waters are contaminated by EDCs discharged from industrial, agricultural, and domestic activities.	0.618
		SA2	Consuming detergents, cleansing agents, or cosmetics, some industries such as textile, paper, cleansing and domestic activities discharge wastewater that contains EDSs.	0.714
		SA3	EDSs in detergents, cleansing agents, cosmetics, etc. may cause feminization in wildlife (fish).	0.661
		SA4	One of the reasons of reproductive disorders in human (e.g., abnormalities in fetus development, testicular dysgenesis syndrome (TDS) in men), is being exposed to EDSs in commonly domestic products such as detergents, cleansing agents, cosmetics, etc.	0.779
		SA5	People may uptake these chemicals via consuming bio-accumulated riverine fish.	0.668
		SA6	People may uptake these chemicals via drinking water exploited from contaminated water resources although the water is treated.	0.676
Risk perception	Risk belief (RB) (α = 0.80)	RB1	I believe that EDSs in commonly domestic products such as detergents, cleansing agents, cosmetics may cause reproductive abnormalities in fish (e.g., feminization of fish).	0.803
		RB2	I believe that EDSs in commonly domestic products such as detergents, cleansing agents, cosmetics may cause reproductive abnormalities in humans.	0.825
	Risk concern (RC) (α = 0.84)	RC1	I concern about those chemicals and their effects on humans although I do not consume riverine fish.	0.805
		RC2	I concern about those chemicals and their effects on humans although I can control my exposure to them.	0.893
Perceived uncertainty (UN) (α = 0.91)		UN1	I am uncertain about the adverse effects on fish and humans because information about EDSs appears in just a few sources.	0.898
		UN2	I am uncertain about the adverse effects on fish and humans because of insufficient attention and warning from scientists.	0.930
		UN3	I am uncertain about the adverse effects on fish and humans because disorders in reproduction can be relevant to other reasons.	0.801
Risk acceptability (RAC) (α = 0.89)		RAC1	I suggest that the level of EDSs from detergents, cleansing agents, cosmetics, etc. in rivers should be controlled for their adverse effects on aquatic life in general, and on riverine fish in particular.	0.912
		RAC2	I suggest that the level of EDSs from detergents, cleansing agents, cosmetics, etc. in rivers should be controlled for their possible effects on humans.	0.858
		RAC3	I suggest that the level of EDSs from detergents, cleansing agents, cosmetics, etc. in rivers should be controlled even though the adverse effects on the health of humans are uncertain.	0.798
Non-diet-related self-protective response (NDSP) (α = 0.79)		SP1	I am thinking of having a frequent health checkup.	0.696
		SP2	I am thinking of having a drinking water checkup.	0.831
		SP3	I am thinking of installing a drinking water filter for my family.	0.722
Diet-related self-protective response ^i^ (DSP)		SP4	I suppose to consume riverine fish less regularly.	

Note: ^i^ “Stand-alone” endogenous variable.

**Table 2:** Model fit indices (^i^ *n* = 331; ^ii^ *n* = 328).

Model	*χ* ^2^	*df*	CMIN/*df*	CFI	TLI	SRMR	RMSEA	PCLOSE
Initial measurement model ^i^	675.0	293	2.3	0.92	0.91	0.06	0.06	<0.010
CLF unconstrained model ^i^	527.1	266	2.0	0.95	0.93	0.05	0.05	0.135
*χ^2^ and df difference*	Δ*χ*^2^ = 147.9	Δ*df* = 27						
Measurement model controlled by experience								
Unconstrained model ^i^	1116.7	605	1.8	0.90	0.89	0.07	0.05	0.396
Measurement weights constrained model ^i^	1097.4	586	1.9	0.90	0.88	0.07	0.05	0.294
*χ^2^ and df difference*	Δ*χ*^2^ = 19.3	Δ*df* = 19						
Measurement model controlled by pregnancy and child status								
Unconstrained model ^i^	1113.6	605	1.8	0.90	0.89	0.07	0.05	0.418
Measurement weights constrained model ^i^	1076.3	586	1.8	0.91	0.89	0.07	0.05	0.434
*χ^2^ and df difference*	Δ*χ*^2^ = 37.3	Δ*df* = 19						
Structural weights model ^ii^	11.6	8	1.4	0.99	0.98	0.03	0.04	0.636
Structural weights model controlled by experience								
Unconstrained model ^ii^	22.1	16	1.4	0.99	0.97	0.04	0.03	0.764
Structural weights constrained model ^ii^	90.2	42	2.1	0.94	0.89	0.11	0.06	0.172
*χ^2^ and df difference*	Δ*χ*^2^ = 68.1	Δ*df* = 26						

**Table 3:** Mean values (*n* = 331).

Construct		Group	Min.–Max. ^i^	Mean ^i^–μ (S.D.)
General awareness of water pollution and reproductive health problems (GA)		Inexperienced	1.4–6.5	5.4 (0.05)
		Experienced	1.2–6.4	5.2 (0.10)
		N_Pr.Ym	1.2–6.5	5.4 (0.06)
		Pr.Ym	1.4–6.4	5.4 (0.07)
Awareness of the pathways of exposure that may affect reproductive health (AP)		Inexperienced	0.5–5.6	3.8 (0.06)
		Experienced	0.9–5.5	3.7 (0.09)
		N_Pr.Ym	0.5–5.6	3.7 (0.06)
		Pr.Ym	0.9–5.4	3.8 (0.08)
Specific awareness of endocrine disrupting surfactants (SA)		Inexperienced	0.1–3.5	1.8 (0.06)
		Experienced	0.1–3.5	1.7 (0.09)
		N_Pr.Ym	0.1–3.5	1.8 (0.06)
		Pr.Ym	0.3–3.5	1.8 (0.08)
Risk perception	Risk belief (RB)	Inexperienced	1.7–6.0	4.1 (0.05)
		Experienced	1.3–5.6	3.8 (0.10)
		N_Pr.Ym	1.3–6.0	4.1 (0.06)
		Pr.Ym	1.7–5.8	4.0 (0.08)
	Risk concern (RC)	Inexperienced	0.7–4.9	4.0 (0.06)
		Experienced	0.8–4.9	4.0 (0.09)
		N_Pr.Ym	0.7–4.9	4.0 (0.07)
		Pr.Ym	2.0–4.9	4.1 (0.07)
Perceived uncertainty (UN)		Inexperienced	0.7–4.1	2.0 (0.06)
		Experienced	0.6–4.1	2.1 (0.09)
		N_Pr.Ym	0.7–4.1	2.1 (0.07)
		Pr.Ym	0.6–4.0	1.9 (0.08)
Risk acceptability (RAC)		Inexperienced	2.6–6.9	5.6 (0.05)
		Experienced	1.5–6.8	5.3 (0.12)
		N_Pr.Ym	2.6–6.9	5.5 (0.06)
		Pr.Ym	1.5–6.9	5.5 (0.09)
Non-diet-related self-protective response (NDSP)		Inexperienced	2.5–6.5	5.5 (0.05)
		Experienced	1.8–6.5	5.2 (0.11)
		N_Pr.Ym	2.3–6.5	5.4 (0.06)
		Pr.Ym	1.8–6.5	5.5 (0.08)
Diet-related self-protective response (DSP)		Inexperienced	1.0–5.0	2.7 (0.07)
		Experienced	1.0–5.0	3.0 (0.11)
		N_Pr.Ym	1.0–5.0	2.8 (0.08)
		Pr.Ym	1.0–5.0	2.7 (0.11)

Notes: ^i^ Min., max., and mean values that accounted for the shared variances of the CLF were imputed from CLF unconstrained model. Inexperienced: lay public; Experienced: experienced public; Pr.Ym: pregnant women and young mothers; N_Pr.Ym: the remaining population.

**Table 4:** Bivariate correlations (*n* = 331).

Construct		Pearson Correlation—r (Spearman’s Rho—r_s_)							
		GA	AP	SA	RB	RC	UN	RAC	NDSP
General awareness of water pollution and reproductive health problems (GA)									
Awareness of the pathways of exposure that may affect reproductive health (AP)		0.331 ** (0.133 *)							
Specific awareness of endocrine disrupting surfactants (SA)		−0.078 (−0.073)	0.014 (0.053)						
Risk perception	Risk belief (RB)	0.249 ** (0.168 **)	0.225 ** (0.223 **)	0.529 ** (0.548 **)					
	Risk concern (RC)	0.337 ** (0.428 **)	0.057 (0.023)	0.184 ** (0.232 **)	0.134 * (0.171 **)				
Perceived uncertainty (UN)		−0.009 (0.047)	−0.084 (−0.118 *)	−0.400 ** (−0.393 **)	−0.317 ** (−0.357 **)	−0.403 ** (−0.498 **)			
Risk acceptability (RAC)		0.427 ** (0.454 **)	0.029 (−0.045)	0.084 (0.056)	0.478 ** (0.362 **)	0.338 ** (0.330 **)	0.066 (0.114 *)		
Non-diet-related self-protective response (NDSP)		0.204 ** (0.201 **)	0.191 ** (0.226 **)	0.357 ** (0.362 **)	0.472 ** (0.486 **)	0.137 * (0.157 **)	−0.210 ** (−0.252 **)	0.520 ** (0.474 **)	
Diet-related self-protective response (DSP)		−0.046 (−0.031)	−0.052 (−0.043)	−0.057 (−0.062)	−0.083 (−0.096)	−0.128 * (−0.131 *)	0.200 ** (0.211 **)	−0.002 (0.020)	−0.068 (−0.119 *)

Note: * Correlation is significant at the 0.05 level (2-tailed); ** Correlation is significant at the 0.01 level (2-tailed).

**Table 5:** Summary of the hypotheses and outcomes (*n* = 328).

Hypothesis	Outcome	Conclusion
H1: Perceived uncertainty (UN) mediates the positive relationships of specific awareness of endocrine disrupting surfactants (SA) with risk belief (RB), risk concern (RC), risk acceptability (RAC), and self-protective response (NDSP & DSP).	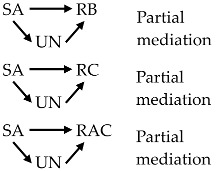	Supported H1 for risk belief and risk concern; partially supported H1 for risk acceptability
	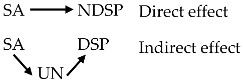	Did not support H1 for self-protective responses
H2: General awareness of water pollution and reproductive health problems (GA & AP) also has direct and positive effects on risk belief (RB) and risk concern (RC).	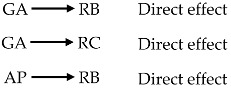	Supported H2
	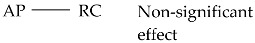	Did not support H2
H3: Perceived uncertainty (UN) plays a mediating role in the positive relationship of risk belief (RB) and risk concern (RC) with risk acceptability (RAC) and self-protective response (NDSP & DSP).	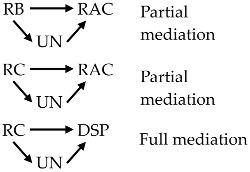	Supported H3
	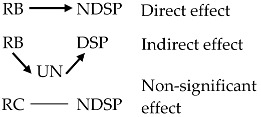	Did not support H3
H4: People distinguished by “experience” and “status of pregnancy and child” differ in the relationships of risk perception (RB) with risk acceptability (RAC) and self-protective response (NDSP & DSP).	Difference by “experience”: RB→RAC (Δ*χ*^2^ = 5.0, Δ*df* = 1; *p* = 0.025) RB→NDSP (Δ*χ*^2^ = 4.6, Δ*df* = 1; *p* = 0.031)	Supported H4
	Non-significant difference by “status of pregnancy and child”	Did not support H4

## Supplementary Materials

The following are available online at http://www.mdpi.com/1660-4601/15/2/296/s1, Table S1: Construct reliability and validity (*n* = 331), Table S2: Construct discriminant validity (*n* = 331), Table S3: The multicollinearity statistics of the independent constructs (*n* = 328), Table S4: The results of normality test for the constructs (*n* = 328).
