# Supplementary material for: The Roles of Three Types of Knowledge and Perceived Uncertainty in Explaining Risk Perception, Acceptability, and Self-Protective Response—A Case Study on Endocrine Disrupting Surfactants
Source: Int J Environ Res Public Health. 2018 Feb 8;15(2):296. doi: 10.3390/ijerph15020296 (PMC5858365; doi:10.3390/ijerph15020296)
Supplement: Supplementary file 1 [file ijerph-15-00296-s001.pdf]

Supplementary material

Table S1. Construct reliability and validity (n = 331).

Latent construct	CR	AVE	MSV	ASV
General awareness of water pollution and reproductive health problems (GA)	0.9	0.6	0.2	0.1
Awareness of the pathways of exposure that may affect reproductive health (AP)	0.9	0.8	0.2	0.1
Specific awareness of endocrine disrupting surfactants (SA)	0.8	0.5	0.4	0.1
Risk belief (RB)	0.8	0.7	0.4	0.2
Risk concern (RC)	0.8	0.7	0.2	0.1
Perceived uncertainty (UN)	0.9	0.8	0.2	0.1
Risk acceptability (RAC)	0.9	0.7	0.3	0.1
Non-diet-related self-protective response (NDSP)	0.8	0.6	0.2	0.1

Table S2. Construct discriminant validity (n = 331).

Latent construct	Pearson correlations							
	GA	AP	SA	RB	RC	UN	RAC	NDSP
General awareness of water pollution and reproductive health problems (GA)	0.742							
Awareness of the pathways of exposure that may affect reproductive health (AP)	0.331**	0.887						
Specific awareness of endocrine disrupting surfactants (SA)	-0.078	0.014	0.707					
Risk belief (RB)	0.249**	0.225**	0.529**	0.814				
Risk concern (RC)	0.337**	0.057	0.184**	0.134*	0.850			
Perceived uncertainty (UN)	-0.009	-0.084	-0.400**	-0.317**	-0.403**	0.878		
Risk acceptability (RAC)	0.427**	0.029	0.084	0.478**	0.338**	0.066	0.857	
Non-diet-related self-protective response (NDSP)	0.204**	0.191**	0.357**	0.472**	0.137*	-0.210**	0.520**	0.755

Notes: * Correlation is significant at the 0.05 level (2-tailed); ** Correlation is significant at the 0.01 level (2-tailed). The square roots of the average variance extracted values were showed in shaded boxes.

Table S3. The multicollinearity statistics of the independent constructs (n = 328).

Independent construct	Tolerance	VIF
General awareness of water pollution and reproductive health problems (GA)	0.68	1.5
Awareness of the pathways of exposure that may affect reproductive health (AP)	0.86	1.2
Specific awareness of endocrine disrupting surfactants (SA)	0.61	1.6
Risk belief (RB)	0.60	1.7
Risk concern (RC)	0.70	1.4
Perceived uncertainty (UN)	0.69	1.4

Table S4. The results of normality test for the constructs (n = 328).

Construct	Group	Minimum	Maximum	Mean	Standard deviation	Skewness			Kurtosis		
						Statistic	S.E.	z-value	Statistic	S.E.	z-value
General awareness of water pollution and reproductive health problems (GA)	Inexperienced	1.4	6.5	5.4	0.05	-2.4	0.16	-15.0	8.9	0.32	27.8
	Experienced	1.2	6.4	5.2	0.10	-2.2	0.25	-8.8	5.4	0.49	11.0
	N_Pr.Ym	1.2	6.5	5.4	0.06	-2.4	0.17	-14.1	7.4	0.33	22.4
	Pr.Ym	1.4	6.4	5.4	0.07	-2.0	0.22	-9.1	7.1	0.44	16.1
Awareness of the pathways of exposure that may affect reproductive health (AP)	Inexperienced	0.5	5.6	3.8	0.06	-0.6	0.16	-3.8	0.8	0.32	2.5
	Experienced	0.9	5.5	3.7	0.09	-0.7	0.25	-2.8	1.0	0.49	2.0
	N_Pr.Ym	0.5	5.6	3.7	0.06	-0.6	0.17	-3.5	0.9	0.33	2.7
	Pr.Ym	0.9	5.4	3.8	0.08	-0.4	0.22	-1.8	0.4	0.44	0.9
Specific awareness of endocrine disrupting surfactants (SA)	Inexperienced	0.1	3.5	1.8	0.06	-0.2	0.16	-1.3	-1.1	0.32	-3.4
	Experienced	0.1	3.5	1.7	0.09	0.3	0.25	1.2	-0.9	0.49	-1.8
	N_Pr.Ym	0.1	3.5	1.8	0.06	-0.1	0.17	-0.6	-1.1	0.33	-3.3
	Pr.Ym	0.3	3.5	1.8	0.08	0.1	0.22	0.5	-1.2	0.44	-2.7
Risk belief (RB)	Inexperienced	1.7	6.0	4.1	0.05	0.2	0.16	1.3	-0.3	0.32	-0.9
	Experienced	1.3	5.6	3.8	0.10	-0.3	0.25	-1.2	-0.3	0.49	-0.6
	N_Pr.Ym	1.3	6.0	4.1	0.06	-0.1	0.17	-0.6	0.3	0.33	0.9
	Pr.Ym	1.7	5.8	4.0	0.08	-0.1	0.22	-0.5	-0.3	0.44	-0.7
Risk concern (RC)	Inexperienced	0.7	4.9	4.0	0.06	-1.4	0.16	-8.8	1.8	0.32	5.6
	Experienced	0.8	4.9	4.0	0.09	-1.4	0.25	-5.6	1.8	0.49	3.7
	N_Pr.Ym	0.7	4.9	4.0	0.07	-1.5	0.17	-8.8	1.7	0.33	5.2
	Pr.Ym	2.0	4.9	4.1	0.07	-0.9	0.22	-4.1	-0.4	0.44	-0.9
Perceived uncertainty (UN)	Inexperienced	0.7	4.1	2.0	0.06	0.6	0.16	3.8	-0.7	0.32	-2.2
	Experienced	0.6	4.1	2.1	0.09	0.4	0.25	1.6	-0.7	0.49	-1.4
	N_Pr.Ym	0.7	4.1	2.1	0.07	0.4	0.17	2.4	-1.0	0.33	-3.0
	Pr.Ym	0.6	4.0	1.9	0.08	0.7	0.22	3.2	-0.1	0.44	-0.2
Risk acceptability (RAC)	Inexperienced	2.6	6.9	5.6	0.05	-1.0	0.16	-6.3	1.3	0.32	4.1
	Experienced	1.5	6.8	5.3	0.12	-1.3	0.25	-5.2	1.1	0.49	2.2
	N_Pr.Ym	2.6	6.9	5.5	0.06	-1.1	0.17	-6.5	1.1	0.33	3.3
	Pr.Ym	1.5	6.9	5.5	0.09	-1.8	0.22	-8.2	3.6	0.44	8.2
Non-diet-related self-protective response (NDSP)	Inexperienced	2.5	6.5	5.5	0.05	-0.8	0.16	-5.0	0.6	0.32	1.9
	Experienced	1.8	6.5	5.2	0.11	-0.8	0.25	-3.2	0.3	0.49	0.6
	N_Pr.Ym	2.3	6.5	5.4	0.06	-0.8	0.17	-4.7	0.4	0.33	1.2
	Pr.Ym	1.8	6.5	5.5	0.08	-1.1	0.22	-5.0	2.1	0.44	4.8
Diet-related self-protective response (DSP)	Inexperienced	1.0	5.0	2.7	0.07	0.1	0.16	0.6	-0.6	0.32	-1.9
	Experienced	1.0	5.0	3.0	0.11	0.0	0.25	0.0	-0.6	0.49	-1.2
	N_Pr.Ym	1.0	5.0	2.8	0.08	0.0	0.17	0.0	-0.6	0.33	-1.8
	Pr.Ym	1.0	5.0	2.7	0.11	0.2	0.22	0.9	-0.6	0.44	-1.4

Note: Inexperienced: lay public; Experienced: experienced public; Pr.Ym: pregnant women and young mothers; N_Pr.Ym: the remaining population.
